# A severe case of neuro-Sjögren’s syndrome induced by pembrolizumab

**DOI:** 10.1186/s40425-018-0429-4

**Published:** 2018-10-22

**Authors:** Jaqueline Ghosn, Alex Vicino, Olivier Michielin, George Coukos, Thierry Kuntzer, Michel Obeid

**Affiliations:** 1 Department of Medicine, Division of Immunology and Allergy, Lausanne University Hospital CHUV, Rue du Bugnon 46, CH-1011 Lausanne, Switzerland; 2 Department of Medical Oncology, Lausanne University Hospital CHUV, Rue du Bugnon 46, CH-1011 Lausanne, Switzerland; 3 Ludwig Institute for Cancer Research, Chemin des Boveresses 155, CH-1066 Epalinges, Switzerland; 4 Vaccination and Immunotherapy Center, Lausanne University Hospital CHUV, Rue du Bugnon 17, CH-1011 Lausanne, Switzerland; 5 Department of Neurology, Lausanne University Hospital CHUV, Rue du Bugnon 46, CH-1011 Lausanne, Switzerland; 6 Medical School Pitié-Salpêtrière, Sorbonne University, 91 Boulevard de l’Hôpital, F-75013 Paris, France

**Keywords:** Checkpoint inhibitors, Immune-related adverse events, Pembrolizumab, PD-1, Neuro-Sjögren’s syndrome

## Abstract

**Background:**

The prevalence of connective tissue disease (CTD) induced by immune checkpoint inhibitors (CPIs) in the absence of pre-existing autoimmunity is unknown.

**Case presentation:**

We report the case of a melanoma patient treated for 8 months with pembrolizumab who developed a subacute ataxic sensory neuronopathy (SNN), including a right trigeminal neuropathy. Salivary gland biopsy showed inflammatory changes suggestive of Sjögren’s syndrome, while brain MRI revealed enhancement of the right trigeminal ganglia. A high level of protein and pleocytosis was found in the cerebrospinal fluid, with negative cultures. Nerve conduction studies revealed the absence of sensory nerve action potentials in the upper and lower limbs and reduced motor responses in the upper limbs, fulfilling criteria for SNN. Blood tests revealed an important inflammatory syndrome, hemolytic anemia, elevation of total IgG levels and the presence of ANA autoantibodies specific to anti-SSA (52 and 60 kd). All these elements were absent before the initiation of the treatment with pembrolizumab. Initially, there was a clinical response following intravenous frontline methylprednisone, but the subacute relapse required the introduction of second-line treatment with intravenous immunoglobulins and then rituximab, which led to a quick clinical improvement.

**Conclusions:**

Herein, we describe the first case of a patient who developed a typical SNN as a complication of severe neuro-Sjögren’s syndrome induced by pembrolizumab treatment.

## Background

Immune checkpoint inhibitors (CPIs) have transformed the prognosis of several advanced malignancies, establishing new standards of care for both adjuvant and metastatic settings. The use of CPIs is associated with a large spectrum of immune side effects, known as immune-related adverse events (irAEs), which may affect every organ [1]. The immunological mechanisms beyond irAEs have not been completely elucidated [1]. Pembrolizumab is a highly selective antiprogrammed cell death 1 (PD-1) humanized monoclonal antibody. The incidence of connective tissue disease (CTD) induced by CPIs treatment is unknown, while that of neurologic irAEs has been reported to be approximately 2.9% [2, 3]. Almost 40 to 50% of these cases are associated with concomitant autoimmune response affecting other organs, such as hepatitis, colitis and hypothyroidism. Frequently, neurological irAEs arise within 6–12 weeks from the initiation of CPIs, although onset may be delayed and even occur after the discontinuation of CPIs [2]. The related neurologic toxicity spectrum involves a wide variety of clinical presentations affecting both the central and peripheral nervous systems, including myopathies, neuromuscular junction disorders, symmetrical length and non-length-dependent peripheral neuropathies (including axonal and demyelinating polyradiculoneuropathies), asymmetric mononeuritis multiplex, cerebellar ataxia and bilateral internuclear ophthalmoplegia [2]. Herein, we report the first case of induced CTD in the form of severe neuro-Sjögren’s syndrome in a patient treated with pembrolizumab.

## Case presentation

A 69-year-old female patient diagnosed with acral lentiginous melanoma of the left foot, pT3a pN2a cM0 R0, stage IIIA, was treated by amputation of the first toe with complete resection. Four years later, she developed histologically confirmed multiple in-transit metastases requiring recurrent excisions. The disease continued to progress, with cutaneous and lymph node metastases. She was enrolled in a clinical trial combining pembrolizumab and T-VEC (*talimogene laherparepvec*, a GM-CSF-expressing oncolytic HSV-1 virus) administered by seven intralesional injections [4]. The patient had no known prior autoimmune disorders and had no neurologic manifestations prior to the CPI treatment.

Four months after the first dose of pembrolizumab, the patient developed several vitiligo lesions, followed by abnormal sensation with tingling and numbness of fingers and hands suggestive of bilateral carpal tunnel syndrome 1 month later. Symptomatic treatment was proposed, but neurological manifestation worsened progressively. At this time, the patient was considered in complete remission and denied having any sicca syndrome, leading to the discontinuation of pembrolizumab after having received eleven administrations.

Between eight and 10 months after the first dose of pembrolizumab, she developed painful tingling sensations in the hands and feet with pseudoathetoid (waver) movements in the arms and hands and a progressive unsteady gait. She was admitted to the hospital for worsening neurologic symptoms and was first seen by the neurologist.

**Fig. 1:**
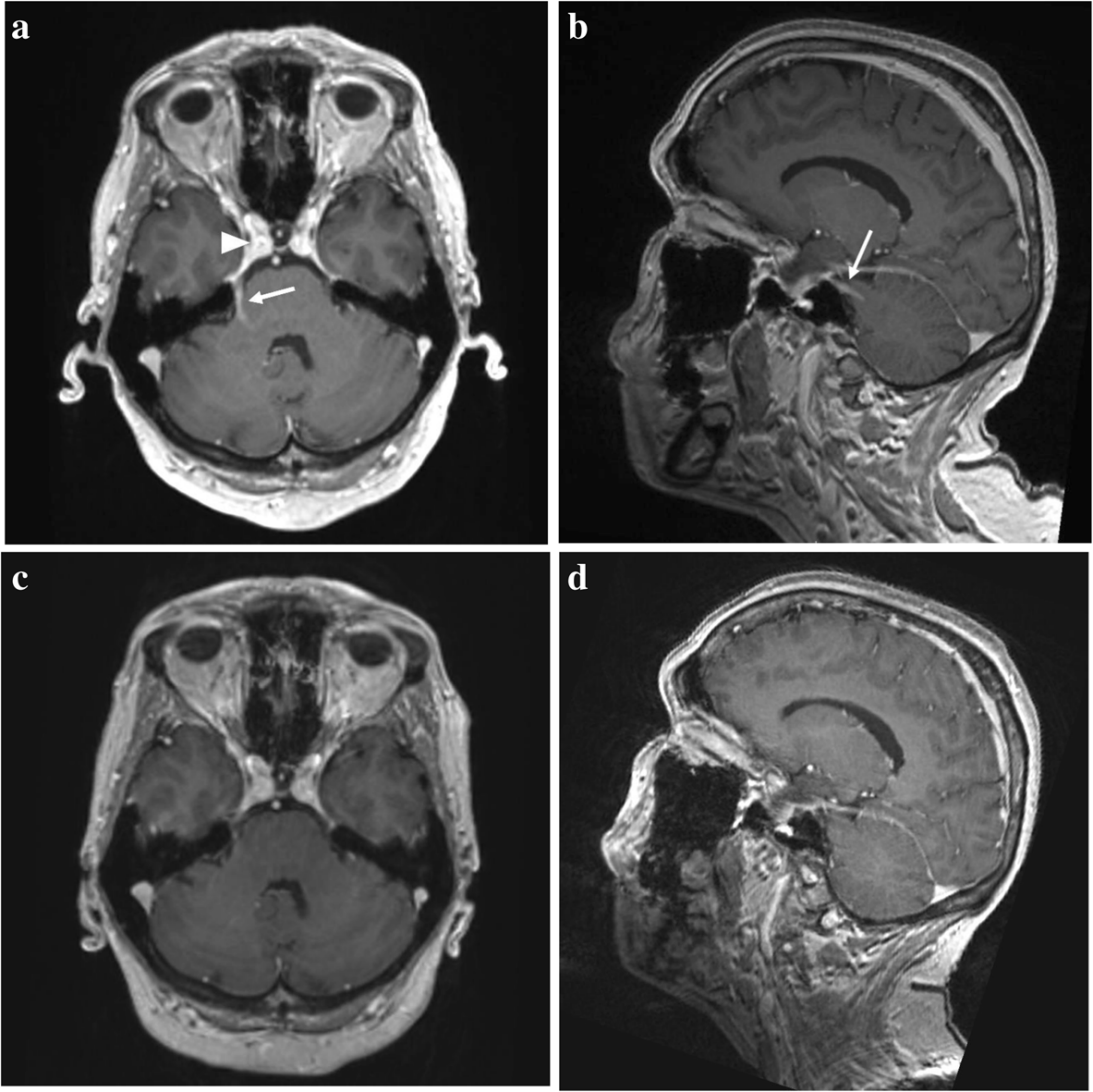
Brain MRI. Sagittal plane (**a**) and axial plane (**b**) showing enhancement of the trigeminal nerve (arrow) from the brainstem to the Gasser’s ganglia (arrowhead). This enhancement disappeared during the treatment [sagittal plane, (**c**), and axial plane, (**d**)]

On examination, she had a loss of sensation of the right face, generalized absence of deep reflexes, and loss of vibration and positional senses in the index and big toes. Nerve conduction studies revealed the absence of sensory nerve action potentials (SNAPs) in the upper and lower limbs and reduced compound muscle action potentials for the median and ulnar nerves but not for the peroneal and tibial nerves. The brain MRI revealed enhancement of the right trigeminal Gasser’s ganglia and its mandibular branch (Fig. 1a and b). The cerebrospinal fluid (CSF) analysis showed a high level of protein (1317 mg/l, normal: < 460) and pleocytosis (92 leukocytes/μl, normal: < 5), with negative cultures, suggestive of aseptic meningitis as CSF PCR testing was negative for herpes-simplex types 1 and 2, varicella-zoster, cytomegalovirus, Epstein-Barr virus, HHV-6 and polyoma JC-virus. Extensive work-up excluded infection with HIV, *Borrelia burgdorferi*, syphilis and hepatitis viruses. A diagnosis of sensory neuronopathy (SNN) was considered probable based on the pattern of her neuropathy with a Camdessanche’s score of 11 (normal: < 6.5) [5]. The patient was at this time restricted to a wheelchair, unable to stand and walk, and the overall neuropathy limitations scale (ONLS) was 9 out of 12 (disability in both arms preventing all function, requires wheelchair to travel 10 m, but able to stand up and walk 1 m) [6]. The patient received intravenous pulses of methylprednisolone (1 g/d) for 5 days and improved progressively over 2 weeks, with an ONLS score passing from 9 to 5 (moderate disability in the upper limbs; walks with unilateral aid for 10 m). She was discharged to a rehabilitation center.

**Fig. 2:**
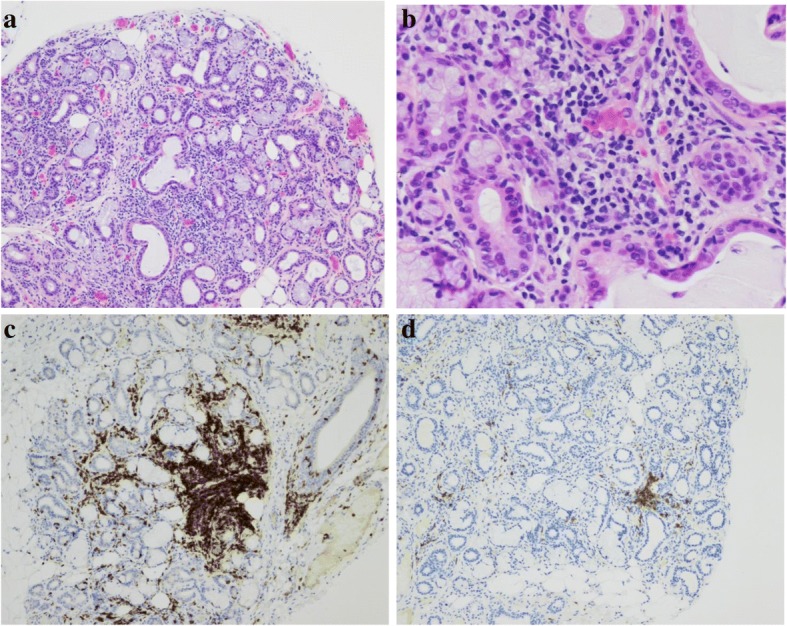
Histopathological examination of a biopsy of the patient’s minor salivary gland. **a** and **b** Hematoxylin- and eosin-colored sections of minor salivary gland biopsy specimens at 100x and 200x magnification, respectively, showing interstitial sclerosis with chronic interstitial inflammation and a focus of ≥50 lymphocytes. **c** and **d** Immunohistochemical staining at 100x magnification with anti-CD3 and anti-CD20 antibody, respectively, with an estimated CD3/CD20 ratio of 80/20

One month later, the patient was readmitted to the hospital for severe ataxic relapse, with an ONLS score of 11, and was restricted to her bed. Investigations were completed by an ^18^*FDG-*PET-CT that found no signs of melanoma recurrence. Blood testing revealed an important inflammatory syndrome with CRP levels at 18 mg/l (normal: < 10); erythrocyte sedimentation rate > 110 mm/h (normal: < 20); hemolytic anemia hemoglobin at 98 g/l (normal: 117 to 157), with haptoglobin at 0.1 g/l (normal: 0.3 to 2.0), LDH at 286 U/l (normal: 135 to 214), total bilirubin at 67 μmol/l (normal: 0 to 21), lymphopenia at 0.8 G/l, an elevation of the total IgG levels to 29.9 g/l, (normal: 7.00 to 14.50) and presence of autoantibodies such as antinuclear antibodies (ANAs): anti-SSA (52 kd at 48 CU, normal level: < 20, and 60 kd at 108 CU, normal level: < 20). A panel of 45 antibodies involved in neurologic paraneoplastic syndromes was negative, including antineuronal (anti-HU and anti-Yo) and antiganglioside antibodies (such as GQ1b). The following analyses were negative or normal: antineutrophil cytoplasmic antibodies (ANCAs): c-ANCA proteinase (PR3), p-ANCA myeloperoxidase (MPO) and atypical ANCA (x or a-ANCA); rheumatoid factor; blood complement C3/C4; creatine kinase; urine spot with proteinuria and creatininuria; creatinemia and serum electrolytes. Of note, ANAs were negative in the serum sample that had been collected prior to the initiation of pembrolizumab. A biopsy of the accessory salivary glands (ASGB) showed abnormal interstitial sclerosis with a focus of > 50 lymphocytes/4 mm^2^ (Chisholm and Mason’s score of 3 out of 4) (Fig. 2). The findings above led to a diagnosis of an induced Sjögren’s syndrome (6 points according to the 2016 ACR/EULAR classification criteria for Sjögren’s syndrome) [7] associated with peripheral nervous system impairment.

After this relapse, an empirical “upfront” treatment with pulses of intravenous methylprednisolone at 1 g/d and immunoglobulins at 0.4 g/kg (both for 5 days) were introduced in combination with acyclovir (which was stopped after negative HSV PCR results in the CSF). In the absence of clinical improvement, a second-line treatment was initiated with cyclophosphamide at 15 mg/kg (one dose) in association with oral prednisone at 60 mg/d. Once the neuro-Sjögren diagnosis was established, cyclophosphamide was replaced with rituximab administered at 375 mg/m^2^ per dose once a month (for 4 administrations at weeks 0, 2, 6 and 10). The decision to change the therapy was made based on the similar efficacy of rituximab and cyclophosphamide for the treatment of the neurologic manifestations of Sjögren’s syndrome and to minimize the risk of T cell suppression and melanoma recurrence.

**Fig. 3:**
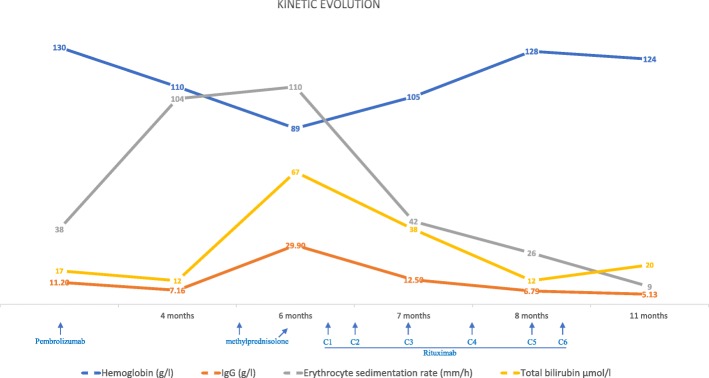
Line graph showing the kinetic evolution of laboratory parameters of the patient including hemoglobin (g/l), total bilirubin (μmol/l), total IgG (g/l) and erythrocyte sedimentation rate (mm/h) (x-axis showing time in months since the initiation of therapy)

The clinical improvement was then rapid, with a progressive amelioration of the ONLS score from 11 to 5. The biological parameters improved in parallel, such as normalization of the levels of hemoglobin, total bilirubin, IgG and erythrocyte sedimentation rate (Fig. 3). The brain MRI showed a marked regression of the enhancement of the trigeminal nerve (Fig. 1c and d).

After six monthly rituximab infusions, we observed the total disappearance of B-cells in a second salivary gland biopsy and normalization of the biological markers, but the patient had persistent neurological deficits, with the absence of deep reflexes, loss of sensation in the distal part of the extremities, and imbalance when walking with bilateral aid for 10 m.

## Discussion and conclusions

Our patient developed subacute neurologic manifestations characterized by painful sensory sensations, pseudoathetoid movements in the arms and hands and a progressive unsteady gait. Deep reflexes and SNAPs were absent, fulfilling the diagnostic criteria of a sensory neuronopathy [5]. SNNs are known for their frequent association with dysimmune disorders including paraneoplastic mechanisms [8]. In our case, the patient was considered in complete remission of her melanoma when sensory manifestations appeared, and repeated work-ups did not suggest a paraneoplastic cause. Among the other dysimmune SNNs, the most frequent association is with Sjögren’s syndrome, accounting for 15–20% of cases in the literature [9].

In our patient, the onset of the neurologic symptoms began 12–14 weeks after CPI initiation, which was slightly delayed compared to the typical neurologic irAEs described in other studies [2], and she presented continued worsening despite the discontinuation of CPIs. This time presentation and outcome are similar to what is observed in complicated neuro-Sjögren’s syndrome and typically require second-line treatment with cyclophosphamide or rituximab. Patients with inflammatory SNN may benefit from immunomodulatory or immunosuppressive treatment if they are administered in a timely manner, and it has been demonstrated that improvement in the disease is possible if patients are treated within 2 months, hence the need for prompt referral to an expert center [10].

Nerve conduction studies, particularly reduced SNAP amplitude, are valuable markers for the diagnosis of SNN [8]. The reduced amplitude of these potentials reflects the sensory neuronal degeneration and is an early parameter for detecting the consequence of the inflammatory changes occurring in the dorsal root ganglia (Gasser’s ganglia is the specific structure dedicated for the face).

In our case, despite a total normalization of the induced inflammation including biological and radiological parameters, the neurological recovery remains partial. One possible explanation is that the delayed treatment of the induced ongoing dysimmune reaction caused irreversible neuronal loss.

Some inflammatory aspects of Sjögren’s syndrome are well characterized, such as the lymphoplasmacytic cell infiltration of the salivary glands [11]. A predominant lymphocytic T-cell infiltration (approximately 80%) was found in our patient, in opposition to the dysimmune mechanisms implicated in the Sjögren’s-associated SNN that have not been fully elicited. However, some immuno-pathological features underlying several forms of neuropathy have been reported by describing the destruction of sensory ganglion cells by lymphocytic infiltration [8]. In our case, except for the ASGB, we do not have other biopsies and in particular no available neuromuscular tissues to assess this point. Other hypotheses have also been mentioned, in particular that the presence of certain “unidentified” antigens primarily responsible for Sjögren’s syndrome could be universally “shared” among the target neural tissues.

Rash, pruritus and vitiligo are the most common skin toxicities reported in CPI treatment, and they occurred early in our patient. The occurrence of vitiligo in melanoma patients receiving immunotherapy is associated with an improved and durable anti-tumor response and better survival [12]. Indeed, the patient has remained in complete remission despite the early discontinuation of immunotherapy. Hematologic irAEs induced by CPIs are relatively uncommon, but cases of autoimmune hemolytic anemia, thrombotic thrombocytopenic purpura and autoimmune thrombocytopenia have been reported [13–15]. In the case of our patient, the origin of the hemolytic anemia could be secondary to the Sjögren’s syndrome, although a direct attribution to pembrolizumab cannot be excluded.

The frequency of CTD fully induced by CPI treatment remains unknown. In contrast, a few reports have estimated the incidence of CTD associated with CPI treatment to be approximately 0.7% (3 out of 447 patients) [16]. However, all three reported patients had pre-existing positive ANAs in serum samples collected prior to CPI initiation, with two patients exhibiting pre-existing anti-SSA (despite the absence of any clinical symptoms for a sicca syndrome or lupus), which was not the case in the serum of our patient, who was free from any type of autoantibodies (absence of ANA, SSA 52 and 60 kd) before CPI initiation. Therefore, we concluded that it was not a decompensation of a pre-existing Sjögren’s syndrome but presumably Sjögren’s syndrome induced by pembrolizumab. To the best of our knowledge, this is the first reported patient who developed Sjögren’s syndrome induced by pembrolizumab treatment without demonstrated pre-existing autoimmunity and complicated by severe neurological manifestations with a disabling sensory neuronopathy. A possible synergetic implication of the concomitant T-VEC injections in the irAEs cannot be formally excluded since this oncolytic herpes virus encoding for GM-CSF may attract dendritic cells to the injection site, which can process the tumor antigens, leading to cytotoxic T cell antigen presentation that may stimulate an immune response. Indeed, it was reported that 1% to 10% of TVEC-injected patients developed anemia and immune-mediated events (such as vasculitis, pneumonia, worsening psoriasis, glomerulonephritis and vitiligo) [17].

This case study raises the question of the indications for autoimmunity baseline screening in asymptomatic patients prior to the initiation of CPIs, including a careful medical history assessment and a baseline autoimmunity laboratory evaluation, to individually assess the autoimmunity risk. Patients with pre-existing disease or asymptomatic autoimmune disorder are at risk of worsening or developing the disease under CPI and should therefore be closely monitored.

A retrospective study published by Menzies et al. sought to explore the safety and efficacy of anti-PD-1 treatment in melanoma patients with pre-existing autoimmune disorders (AIDs) and a history of irAEs during ipilimumab treatment. Of the 119 patients included, 52 had a pre-existing autoimmune disorder, and 20 (38%) had a flare of the autoimmune disorder requiring immunosuppression [18]. In case of suspected induced AIDs, the assessment of the kinetics of respective autoantibody titers can be helpful for the diagnosis and may aid in the early recognition and management of treatment-related adverse events [19].

In conclusion, we have demonstrated here that a severe disabling neuropathy could be associated with neuro-Sjögren’s syndrome induced by pembrolizumab, underlining the fact that a particular type of CTD can be de novo induced in patients with no prior clinical or laboratory evidence of autoimmune disorders. Clinicians are challenged to rapidly identify these complications and promptly initiate immunosuppression or immunomodulatory treatment, which is essential to optimize clinical outcome if administered in a timely manner. To date, however, the optimal duration of immunosuppressive treatment and the risks of late serious toxicities remain unknown.

## Abbreviations

AIT: Autoimmune toxicity
ASGB: Accessory salivary glands biopsy
CPIs: Checkpoint inhibitors
CSF: Cerebrospinal fluid
CTD: Connective tissue disease
ESR: Erythrocyte sedimentation rate
irAEs: Immune-related adverse events
PD-1: Programmed death 1 receptor
PD-L1: Programmed death-ligand 1
SNAPs: Sensory nerve action potentials
SNN: Sensory neuronopathy
